# Ovarian vein thrombosis

**DOI:** 10.11604/pamj.2015.21.251.6908

**Published:** 2015-08-06

**Authors:** Amel Achour Jenayah, Sarra Saoudi, Fethia Boudaya, Ines Bouriel, Ezzeddine Sfar, Dalenda Chelli

**Affiliations:** 1 Department A of Gynecology and Obstetrics, Center of Maternity and Neonatology of Tunis, Tunisia

**Keywords:** Ovarian vein thrombosis, postpartum, pregnancy

## Abstract

Ovarian vein thrombosis (OVT) is a rare cause of abdominal pain that may mimic a surgical abdomen. It is most often diagnosed during the postpartum period. In this report, we present four cases of postoperative ovarian vein thrombosis. The complications of OVT can be significant, and the diagnosis relies on a careful examination of the radiographic findings. It can occur with lower quadrant abdominal pain, especially in the setting of recent pregnancy, abdominal surgery, pelvic inflammatory disease, or malignancy. Diagnosis can be made with confidence using ultrasound, computed tomography or magnetic resonance imaging. Treatment of ovarian vein thrombosis is particularly important in the post-partum patients, with anticoagulation therapy being the current recommendation.

## Introduction

Postpartum ovarian vein thrombosis (POVT) is a rare puerperal complication, with an incidence of 1/600 and 1/2000 deliveries. It occurs in 0.05% of all pregnancies that results in live births [1]. Ovarian vein thrombosis is a rare complication which arises classically in the post-partum. Ovarian vein is the commonest vein involved in puerperal pelvic thrombophlebitis [1]. It occurs in non pregnant patients. A few cases has been reported [1, 2]. **Objectif:** our purpose is to present the clinical features and the evolution of four patients admitted in the service A of the Center of maternity and neonatology of Tunis for essentially abdominal pain (three cases) and to whom the radiological examinations had revealed a thrombosis of the ovarian vein.

## Patient and observation

### Case report 1

**Figure 1:**
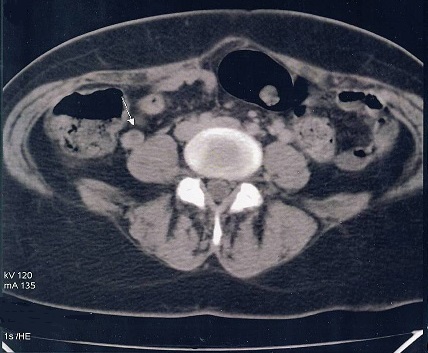
Thrombosis of the right ovarian vein showed on spiral CT

A 31 years old woman, (gravida 1, para 1) reported right-sided abdominal pain approximately 48 hours after her caesarean section. She was a non-smoker and had no significant past medical, surgical, or family history. Clinical evaluation on physical examination, demonstrated a minimal amount of vague lower abdominal pain with a temperature of 38.2°, leukocytosis of 12,500 cells/µL (normal, 4500-10,000 cells/µL) and CRP level of 420. The patient was observed during the night, and when her pain did not resolve the next morning, pelvic and abdominal ultrasound were done and were normal. Abdominal computed tomography (CT) with intravenous contrast was ordered to evaluate for a surgical source for her persistent pain, such as appendicitis. This scan revealed a right ovarian vein thrombosis (Figure 1).

A hypercoagulability work-up revealed no abnormalities. The patient started on anticoagulation therapy for her ovarian vein thrombus. Heparin was discontinued, and one year course of oral warfarin was initiated. The patient was discharged home 5 days later in good condition. At follow-up one year later, she had not experienced recurrence of her symptoms while being maintained on therapeutic anticoagulation.

### Case report 2

A 30 years old woman, (gravida 3, para 2), presented to the emergency department complaining of cough, minor haemoptysis, and a temperature of 38,3°. She was 9 days postpartum from an uncomplicated caesarean section. She was a non-smoker who had no significant past medical, surgical, or family history. Clinical evaluation the patient's physical examination was remarkable for tachycardia to 108, an oxygen saturation of 94% on 2 liters oxygen supplementation via nasal cannula, clear lungs, and abdomen that was minimally tender to palpation in the deep pelvis. A pelvic examination performed by the obstetrician revealed a non tender uterus and normal brown-red lochia. The white blood cell count was 15,700 cells/µL and CRP level of 177. Pelvic and abdominal ultrasound were done and were normal.

The presence of haemoptysis raised concern about the possibility of pulmonary embolism, and a spiral CT scan of the chest confirmed a right lower lobe pulmonary embolism. Doppler ultrasound of the legs did not reveal deep venous thrombosis. Additional evaluation with an abdominopelvic CT was then obtained, which demonstrated right ovarian vein thrombosis. Management Anticoagulation therapy with heparin was initiated along with intravenous antibiotics for pulmonary embolism and systemic sepsis resulting from the right ovarian vein thrombosis. The patient's leukocytosis and fever resolved after 48 hours of intravenous antibiotic therapy. A hypercoagulability work-up revealed no abnormalities. Heparin was discontinued, and one year course of oral warfarin was initiated. The patient was discharged home 20 days later in good condition. At follow-up one year later, she had not experienced recurrence of her symptoms while being maintained on therapeutic anticoagulation.

### Case report 3

A 36 years old woman, (gravid 10, para 7) reported right-sided abdominal pain approximately 17 days after her caesarean section. She was a non-smoker and had no significant past medical, surgical, or family history. Clinical evaluation on physical examination, demonstrated a moderate abdominal pain with a temperature of 38,5°, leukocytosis of 14,800 cells/µL and CRP level of 320. Pelvic and abdominal ultrasound were done and were normal. Abdominal computed tomography (CT) with intravenous contrast was ordered to evaluate for a surgical source for her persistent pain. It revealed a right ovarian vein thrombosis.

A hypercoagulability work-up revealed no abnormalities. The patient started on anticoagulation therapy for her ovarian vein thrombus. Heparin was discontinued, and a one year course of oral warfarin was initiated. The patient was discharged home 12 days later in good condition. At follow-up one year later, she had not experienced recurrence of her symptoms while being maintained on therapeutic anticoagulation.

### Case report 4

**Figure 2:**
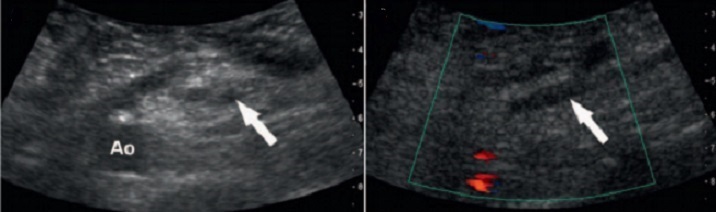
Thrombosis of the right ovarian vein showed on the doppler ultrasonography

A 43 years old woman, (gravida 3, para 3), with no medical history. She consults 10 days after her hysterectomy for abdominal pain with a temperature of 38,5°, leukocytosis of 16.880 cells/µL and CRP level of 280. Pelvic and abdominal ultrasound were done and were normal (there is no textilome neither abscesses). The diagnosis of appendicitis was eliminated. A Doppler ultrasound confirmed the diagnosis of thrombosis of the right ovarian vein (Figure 2). Immunological assessment was normal (S protein, C protein, Leiden V factor were normal). The patient started on anticoagulation therapy for her ovarian vein thrombus. Heparin was discontinued, and one year course of oral warfarin was initiated. The patient was discharged home 11 days later in good condition. At follow-up one year later, she had not experienced recurrence of her symptoms while being maintained on therapeutic anticoagulation.

## Discussion

The cases presented here are interesting in terms of the rarity of this entity and the succefull treatment undertaken. Apart from several reports on ovarian vein thrombosis; it is an uncommon condition that has been estimated to occur in 0.05% of all pregnancies that results in live births [1].

This condition is classically a puerperal process, but it may also arise in non puerperal settings such as endometritis, pelvic inflammatory disease, malignancy, thrombophilia, inflammatory bowel disease, and pelvic and gynecologic surgeries [3–5]. This article presents here three cases of thrombosis ovarien vein in the post partum and one case in non pregnant patient.

Pregnancy is a hypercoagulable state in which there is increased platelet adhesion and decreased fibrinolysis coupled with increasing levels of factors I, II, VII, VIII, IX, and X [1]. Erythrocyte mass increases approximately 20% to 30%, while plasma volume increases by 40% to 50%. This relatively hemodilutional state during pregnancy serves to limit maternal blood loss at delivery [4].

Several physiologic and anatomic factors predispose the right ovarian vein to thrombosis. Right ovarian vein was implicated in our patients. Studies [6] have shown that 80% of cases affect the right ovarian vein, while the left ovarian vein and both ovarian veins are involved in 6% and 14% of cases, respectively. The increase involvement of the right ovarian vein may be explained by the compression of the inferior vena cava and right ovarian vein due to dextrorotation of the uterus during pregnancy. Other contributory factors are antegrade flow of blood in the right ovarian vein favouring bacterial infection, in contrast to retrograde blood flow in the left ovarian vein. Also there are multiple incompetent valves in the right ovarian vein [6]. A high index of suspicion for ovarian vein thrombosis is needed to establish the diagnosis, regardless of the setting. During pregnancy, the diameter of the ovarian vessels increases due to increased blood flow and hormonal changes, resulting in substantially increased pressure on both the vessel walls and the valves within the veins. This increased pressure at the valves results in venous incompetence, compounding venous stasis in the pelvis [7]. In addition, the gravid uterus may undergo physiologic dextrorotation, potentially compressing the already engorged right ovarian vein. Also, the right ovarian vein enters the inferior vena cava at an acute angle, making it more susceptible to compression, whereas the left typically enters the left renal vein at a right angle. Finally, in the postpartum period, blood flow in the right ovarian vein is antegrade as compared with retrograde in the left vein, potentially predisposing to right-sided thrombosis [8, 9]. It may also complicate group A and B streptococcal infection of the vagina and endometrium resulting in endothelial injury.

Ovarian vein thrombosis often has a vague and variable presentation, and a high index of suspicion is required to make the diagnosis. Presentation classically, ovarian vein thrombosis arises in the first 7 days postpartum. In the postpartum period, up to 80% of patients will present with fever, but only half will experience right lower quadrant abdominal pain [10]. Importantly, many patients will have nonspecific symptoms, including malaise, vague diffuse abdominal pain, or shortness of breath. In rare cases, a mass may be palpable, but this is an unusual finding [11, 12]. In our cases, our patients complained with abdominal pain and temperature. Diagnosis of POVT can be made by Doppler sonography, contrast enhanced computerized tomography scan, and magnetic resonant angiography [13]. The latter has 100 percent sensitivity and specificity.

The accuracy of ultrasound in confirming the diagnosis of ovarian vein thrombosis is highly operator dependent, which should limit its role in obtaining the initial diagnosis [14]. Additionally, overlying bowel gas may limit visualization in ultrasound, which oftentimes causes the operator to confuse ovarian vein thrombosis with the appendix or hydroureter. However, ultrasound may have a role for follow-up imaging in patients previously diagnosed with the condition.

Abdominopelvic CT scan with intravenous contrast has a sensitivity and specificity nearing 100% in some studies and should be considered the initial investigative step because it is readily obtainable and is more cost-effective than magnetic resonance imaging (MRI) [14].

On CT, differentiation of the thrombosed ovarian vein from the appendix can be difficult; visualization of a tubular retroperitoneal mass with central low attenuation extending cephalic to the inferior vena cava is characteristic of ovarian vein thrombosis. MRI can provide additional information in patients with a strong clinical suspicion for the diagnosis but equivocal CT findings or in patients with a contrast dye allergy [13].

Laparoscopy is also a useful diagnostic method. Appendicitis, endometritis, pyelonephritis, adnexal torsion/abscess, which are common causes of lower abdominal pain in the puerperium, should be considered as differential diagnosis [15]. Torsion of a pedunculated uterine fibroid should be included in the list of differential diagnosis of POVT.

Complications of ovarian vein thrombosis most frequently occur in the postpartum period, the most serious being systemic sepsis and pulmonary embolism. The risk for developing complications of ovarian vein thrombosis correlates with the clinical setting in which the condition arises. In a small review involving 6 patients diagnosed with ovarian vein thrombosis in the setting of a malignant solid tumor, none developed pulmonary embolism or localized abdominal pain. Additionally, the study showed that several patients had resolution of the ovarian vein thrombosis during follow-up without anticoagulation therapy. A second study revealed that 40 out of 50 patients (80%) undergoing total abdominal hysterectomy with bilateral salpingo-oophorectomy and retroperitoneal lymph node dissection for carcinoma had documented ovarian vein thrombosis on postoperative surveillance CT scanning. None of these patients had abdominal or pulmonary symptoms to suggest any complications from the ovarian vein thrombus, and none were treated with anticoagulation. Furthermore, Bates [13] in his study that included both male and female patients found that the development of gonadal vein thrombosis after diverticulitis, inflammatory bowel disease, perforated appendicitis, and pseudomembranous colitis more commonly occurred on the left side. Although the body of literature is still quite small, it appears that in the setting of malignancy or recent pelvic surgery, observation of ovarian vein thrombosis on either side without anticoagulation is appropriate. Complications of ovarian vein thrombosis are more common in the postpartum period. Extension of the clot into the inferior vena cava or renal veins, acute ureteral obstruction, sepsis, pulmonary embolism, and death have been documented as a consequence of the ovarian vein thrombosis in the postpartum period [6, 9].

The incidence of pulmonary embolism after puerperal ovarian vein thrombosis varies widely, ranging from 0,15 to 0,33% in the highest reports, with a resultant mortality rate up to 4% [14]. Up to one second of postpartum cases may result in pulmonary embolism, and mortality estimates approach 5% [16]. There is no clear consensus in the literature regarding optimal treatment of this condition. Bates [13], in his randomized study demonstrated no episodes of pulmonary embolism and no outcome differences among 14 women diagnosed with septic pelvic thrombophlebitis who were randomized to intravenous antibiotics alone (n = 8) or intravenous antibiotics plus heparin (n = 6).

However, because of the increased risk of a potentially lethal pulmonary embolism, most reviews support treatment of postpartum ovarian vein thrombosis with intravenous anticoagulation. Most patients will present with fever, and antibiotic therapy is typically initiated for the presumptive diagnosis of endometritis, prior to securing the true diagnosis of ovarian vein thrombosis. The duration of anticoagulation therapy is controversial.

Resolution of ovarian vein thrombosis has been documented after only 7 to 14 days of therapy [9]. Others have shown that ovarian vein thrombosis may not resolve with short anticoagulation therapy, and 3 to 6 months of anticoagulation is indicated until there is radiologically confirmed resolution of the thrombus. An association of puerperal ovarian vein thrombosis with inherited hypercoagulability disorders has been noted, which may predispose these patients to ovarian vein thrombosis.

Management approach of POVT may be medical or surgical treatment, with both recording similar success rate. The main approach to medical treatment involves the use of anticoagulant. The inclusion of broad spectrum antibiotics for 7 to 10 days has also been recommended. While the place of surgery in the initial management of POVT is controversial, some clinicians prefer surgery for complicated cases associated with free floating thrombosis, recurrent pulmonary emboli in spite of medical treatment, and contraindication to anticoagulant use [6]. Mortality rate of 52% was recorded among untreated cases. However, with the use of anticoagulant, the mortality among treated cases reduced from 25% to 5%. The four patients managed had anticoagulant therapy. Recurrence of POVT is low in subsequent pregnancy. But for patients with underlying hypercoagulable state, anticoagulant prophylaxis is recommended in future pregnancies.

## Conclusion

Although ovarian vein thrombosis is uncommon, it should be included in the differential diagnosis for postoperative women presenting with vague abdominal symptoms given the potentially fatal outcome associated with this condition. The published literature clearly indicates that complications such as pulmonary embolism, sepsis, and thrombus extension are more likely to occur in the puerperal setting and thus seems to support use of anticoagulation therapy, given the difficulties in predicting the development of these complications.
